# Scorbutus—Scurvy

**Published:** 1857-08

**Authors:** Thos. A. Hill

**Affiliations:** Reading, Ill.


ARTICLE VI.—*Scorbutus—Scurvy.* By THOS. A. HILL, M. D., Reading, Ill.

This disease is one that has never found a place in the catalogue of diseases for which I have prescribed until recently. I am not aware that it prevails in any other part of the State; yet, as the same cause—or at least what I conceive to be the cause—exists over a large portion of the State, I presume the same effect follows. I have always regarded scurvy as a disease of rare occurrence to the Western practitioner; consequently, I never expected it would occupy so large a share of my time as it has for the last three months.

The disease appeared here in the early part of February, since which there have been a great many cases; and, in fact, cases of scurvy have outnumbered those of all other diseases.

In many it appeared in a violent form; in others, again, with no other symptom than languor, spongy and hemorrhagic gums, slight œdema of the feet, and an eruption resembling petechia on the legs and arms.

The cause of the prevalence of the disease here is obviously owing to a long continued abstinence or deprivation of fresh provisions, and a due quantity of acescent food, assisted by the intense cold of last winter. Owing to the scarcity and consequent price of fresh meat and vegetables, many have subsisted for several months almost exclusively on salted food.

Women during lactation appear to contract the disease more readily than others; a large proportion of the cases here were of this kind. In all cases, both male and female, there existed a debilitating cause. I saw no cases where the patient was healthy previous to the premonitory symptoms of scurvy.

The disease comes on with lassitude, complexion pale and bloated, spongy and hemorrhagic gums, and a feeling of stiffness of the flexor muscles of the legs, with slightly accelerated pulse. As the disease progresses, the gums become much swollen, and present a livid appearance, and sloughing off. Extremities œdematous, tense, glassy, and covered with livid and purple spots and painful. The muscular powers are now so prostrated that the patient can scarcely maintain the erect position.

The treatment in all cases has been simple, and so far successful. The first thing to be observed is to remove the existing cause. Let the patient be well provided with vegetable food, amongst which I find potatoes, turnips and cabbages cooked with vinegar preferable to all others. In my opinion, these articles eaten uncooked, or cooked, and eaten with vinegar, is all-sufficient in a large proportion of cases. Where medicine is required, I generally prescribe the following tonic preparaion:

℞ Tinct. Columbo, Tinct. Gentian, Tinct.
Quassia, Tinct. Rhei, - - - a a ℥i.

Mix; take a tablespoonful four or five times a day. This, together with a free use of lemon juice or citric acid, are about all the internal remedies that I have made use of. For ulceration or sloughing of the gums, I apply the Nit. Argent. until the gums are thoroughly cauterized.

